# Myeloid-related protein 8/14 in plasma and serum in patients with new-onset juvenile idiopathic arthritis in real-world setting in a single center

**DOI:** 10.1186/s12969-022-00701-x

**Published:** 2022-06-16

**Authors:** Paula L. Keskitalo, Salla M. Kangas, Sirja Sard, Tytti Pokka, Virpi Glumoff, Petri Kulmala, Paula Vähäsalo

**Affiliations:** 1 PEDEGO Research Unit, University of Oulu, Oulu, Finland; 2 Department of Pediatrics, Oulu University Hospital, Kajaanintie 50, 90220 Oulu, Finland; 3 Medical Research Center, Oulu University Hospital and University of Oulu, Oulu, Finland; 4 Research Unit of Biomedicine, University of Oulu, Oulu, Finland

**Keywords:** Biomarker, Calprotectin, Juvenile idiopathic arthritis, S100A8/A9, MRP8/14

## Abstract

**Objective:**

The aim of this study was to analyze the usefulness of myeloid-related protein 8/14 (MRP8/14) in the prediction of disease course in a real-world setting for patients with new-onset juvenile idiopathic arthritis (JIA), to identify the relationship between MRP8/14 and disease activity using the physician’s global assessment of disease activity (PGA), and determine whether the MRP8/14 levels measured in serum and plasma are equally useful.

**Methods:**

In this prospective follow-up study, 87 new-onset non-systemic JIA patients were studied. Blood and synovial fluid samples were collected prior to any antirheumatic medication use. MRP8/14 was measured from serum (S-MRP8/14), plasma (P-MRP8/14), and synovial fluid samples using ELISA.

**Results:**

The baseline MRP8/14 blood levels were significantly higher in patients using synthetic antirheumatic drugs than in patients with no systemic medications at 1 year after diagnosis in serum (mean 298 vs. 198 ng/ml, *P* < 0.001) and in plasma (mean 291 vs. 137 ng/ml, *P* = 0.001). MRP8/14 levels at the time of JIA diagnosis were higher in patients who started methotrexate during 1.5-year follow-up compared to those who achieved long-lasting inactive disease status without systemic medications (serum: mean 298 vs. 219 ng/ml, *P* = 0.006 and plasma: 296 vs. 141 ng/ml, *P* = 0.001). P-MRP8/14 was the most effective predictive variable for disease activity (by PGA) in linear multivariate regression model (combined to ESR, CRP, leukocytes, and neutrophils), whereas S-MRP8/14 was not significant.

**Conclusion:**

Blood MRP8/14 levels at baseline seem to predict disease course in new-onset JIA patients. P-MRP8/14 might be better than S-MRP8/14 when assessing disease activity at the time of JIA diagnosis.

## Introduction

Juvenile idiopathic arthritis (JIA) is a heterogeneous group of diseases characterized by arthritis of unknown etiology persisting at least 6 weeks and with onset before 16 years of age [1]. The disease course of JIA can be aggressive and require rapid medical intervention, or it can be milder and sometimes even self-limiting. Due to the disease’s heterogeneity, it is a major challenge to distinguish those new-onset JIA patients who require rapid treatment interventions to avoid long-term disability from those who do not need systemic treatment and avoid exposing them to the medication’s potential side effects. New reliable prognostic markers are needed to better predict the course of the disease and achieve this goal, as well as to facilitate the future aim of individualized treatment options.

Biomarkers are crucial components of personalized medicine used to measure and demonstrate changes that correlate with disease manifestations or that have diagnostic or prognostic benefits [2]. The assessment of the actual inflammatory activity levels of JIA patients and changes to those levels is challenging in clinical practice because of the disease’s multidimensional nature. Commonly used inflammatory laboratory parameters such as C-reactive protein (CRP) and erythrocyte sedimentation rate (ESR) are biomarkers that reflect systemic rather than local inflammatory processes. Hence, the need to identify more specific inflammatory markers of local inflammation (e.g., synovial inflammation in JIA) has led to the identification of new molecules such as myeloid-related protein 8 and 14 complex (MRP8/14), also known as calprotectin [3].

MRP8 and MRP14 are intracellular calcium-binding proteins expressed by monocytes and granulocytes, and they exist as heterodimer complexes in the cytosol. In synovial inflammation, the infiltrating phagocytes secrete the complex, and it acts as a ligand of the Toll-like receptor 4 [4–7].

Several studies have proposed that disease activity in JIA correlates better with serum MRP8/14 levels than with the systemic inflammatory markers CRP and ESR [8–10]. Various studies have also demonstrated that MRP8/14 levels might be a good predictor of disease flares [7, 11–14] and that it could predict responses to systemic treatment [7, 12, 15–17], disease progression, and treatment escalation in JIA [18]. The opposite results have also been reported [19, 20].

Previous studies of MRP8/14 have analyzed either serum or plasma samples. Recently, Nordal et al. found a stronger association between disease activity and calprotectin measured in plasma than between disease activity and calprotectin measured in serum in adult patients with rheumatoid arthritis [21]. To our knowledge, there are no comparative studies of MRP8/14 measured in both serum and plasma in JIA patients.

Thus, the aim of our study was to evaluate whether blood MRP8/14 levels can be used to predict disease course in a real-world population of new-onset non-systemic JIA patients and to analyze whether the MRP8/14 levels measured in serum and plasma were equally relevant in clinical use. We also studied whether MRP8/14 levels reflect clinical disease activity in JIA onset better than other laboratory parameters do.

## Methods

### Patients

In a prospective population-based follow-up study, we examined 135 consecutive patients presenting with non-systemic new-onset arthritis under the age of 16 years. Patients were recruited between October 2011 and November 2014 at a pediatric rheumatology outpatient clinic at Oulu University Hospital. This facility is the tertiary hospital to which primary care physicians from the Oulu University Hospital district refer all their patients with suspected JIA. The mean population of children under 16 years of age in this area was 93,000 during the study years. The JIA diagnostic criterion established by International League of Associations for Rheumatology (ILAR) [1] was fulfilled in 108 patients. Twenty patients were excluded because the research laboratory staff was not available to handle samples. Postponing sampling was not an option because samples had to be collected before starting medication. One family refused to participate. Finally, 87 patients with JIA were eligible for this study.

### Data collection

The clinical patient data included medical history and a physical examination of the following: number of joints with active disease (active joint count), physician’s global assessment of disease activity (PGA), parent or patient assessments of overall well-being (data available for 53 patients), and functional ability measured by the Childhood Health Assessment Questionnaire (CHAQ) (data available for 39 patients). Clinical data were also collected, and routine laboratory parameters were measured at scheduled appointments when appropriate.

We used the modified definition of the term “inactive disease” given by Anink et al. [12]: no active arthritis, no systemic features, no uveitis, normal ESR (≤ 20 mm/h), and PGA indicating no disease activity (score ≤ 10 on a scale of 0 to 100 mm). The modified criteria include an increased threshold of PGA compared to the Wallace criteria for inactive disease [22]. We chose this definition because of the difficulty of setting the PGA at zero when patient or parent still reports symptoms such as pain and stiffness despite no objective findings of active joints.

### Evaluation of disease course

To predict disease course and the need for disease-modifying antirheumatic drugs (DMARDs), we divided patients into three groups according to need for the systemic treatment at 1 year after JIA diagnosis. We compared the baseline biomarker levels in patients who achieved long-lasting inactive disease status (i.e., for at least 6 months) without DMARDs and patients who started methotrexate (MTX) treatment within 8 months of baseline. We also assessed the ability of the biomarkers to predict disease activity during 1.5-year follow-up. Inactive disease as defined by Wallace [22] involves only one time point. Our study’s endpoint constitutes a stricter goal than the American College of Rheumatology (ACR) pediatric criteria for JIA responses used in several previous studies [11, 12, 15, 16, 23].

### Serological samples and measurements

Serum samples were available from 87 patients, plasma samples were available from 72 patients, and synovial fluid samples were available from 48 patients. We collected 49 serum samples and 37 plasma samples from healthy controls.

Serum, plasma, and synovial fluid samples for MRP8/14 were and centrifuged within 2 hours of collection. Plasma samples were pipetted from a BD Vacutainer® CPT™ containing buffered sodium citrate after centrifugation. The samples were aliquoted and stored at − 80 °C until analysis.

MRP8/14 levels in serum, plasma, and synovial fluid were measured with the enzyme-linked immunosorbent assay (ELISA) using the human calprotectin ELISA kit (Hycult Biotech) according to kit protocol. For the ELISA, serum and plasma samples were diluted to 1:60, and synovial fluid samples were diluted to 1:200 with the dilution buffer included in the kit. For plasma MRP8/14 samples, that were pipetted from BD Vacutainer® CPT™ tubes and slightly diluted, we used a correction coefficient of 1.125 to compensate for the dilution. When the MRP8/14 result was below the stated detection range, we gave it a value of 50% of the lower detection limit: serum 2.0 ng/ml, plasma 1.2 ng/ml and synovial fluid 29.1 ng/ml. Absorbance was measured at 450 nm using the Wallace Victor2 1420 multilabel counter. Quantitative analysis of the samples was performed using a four-parameter logistic curve fit, and data were analyzed using MyAssays Analysis Software Solutions (MyAssays: http://www.myassays.com).

Inflammatory markers, including leukocyte, neutrophils, and ESR and CRP levels were measured as part of the clinical assessment. Synovial fluid samples from those patients who received intra-articular steroid injections were collected whenever possible, and leukocyte counts and MRP8/14 concentrations were assayed.

### Controls

As controls, we collected blood samples from 49 healthy children (aged 1 to 16 years, 52% males) with no acute infection within the previous 2 weeks and no history of autoimmune or inflammatory diseases. Most of them were healthy children who underwent a minor surgical procedure and had a blood sample taken at the same time, and the rest were voluntary healthy children who came only for blood sampling.

### Statistical analysis

Descriptive statistics are presented as absolute frequencies and both median and interquartile range (IQR) and mean and standard deviation (SD), when appropriate. Correlations were analyzed using Pearson’s correlation coefficients (r). Linear regression was used to evaluate the associations between PGA and inflammatory parameters. Differences between groups were analyzed using a t-test or one-way ANOVA, and post-hoc analyzes were performed using Tukey’s honest significant difference test when normally distributed. *P* values of less than 0.05 in two-tailed tests were considered statistically significant. All data were analyzed using SPSS Statistics Software for Windows version 27 (IBM Corp., Armonk, New York, USA).

## Results

### Characteristics of patients and controls

**Fig. 1:**
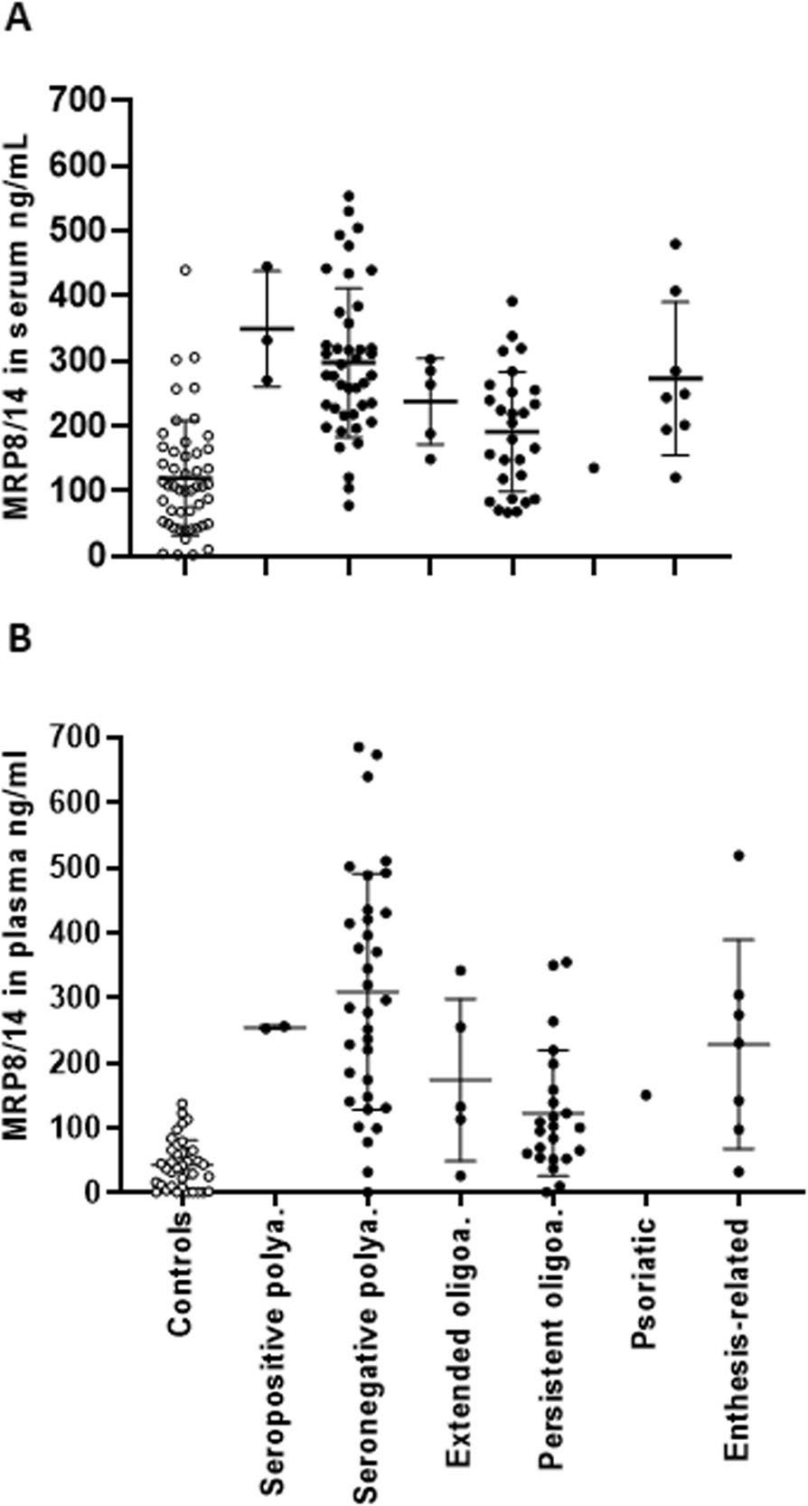
Myeloid-related protein 8/14 levels in the control children and in the juvenile idiopathic arthritis patients. Myeloid-related protein (MRP) 8/14 measured at JIA diagnosis in serum (S-MRP8/14) (**A**) and in plasma (P-MRP8/14) (**B**). Each symbol represents the value of a given parameter for an individual patient. Results are given in terms of mean and standard deviation (SD)

The baseline characteristics of the 87 JIA patients are presented in Table 1. In JIA patients, the mean serum level of MRP8/14 (S-MRP8/14) was 257 ng/ml (SD 115), and the mean level of plasma MRP8/14 (P-MRP8/14) 229 ng/ml (SD 168) (Fig. 1). Figure 1 also includes the control group, but it was not used in the comparison of MRP8/14. Compared to patients with persistent oligoarthritis, patients with seronegative polyarthritis had significantly higher S-MRP8/14 levels [mean 297 ng/ml (115) vs. 189 ng/ml (91); *p* < 0.001] and higher P-MRP8/14 levels [mean 309 ng/ml (182) vs. 124 ng/ml (95); *p* < 0.001]. There were no significant differences in serum or plasma MRP8/14 levels among the other JIA categories. MRP8/14 concentrations were significantly higher in synovial fluid (SF) [mean 1292 ng/ml (409)] than in blood samples (*p* < 0.001 for both serum and plasma) at the time of diagnosis. SF-MRP8/14 concentrations did not differ among JIA categories. SF-MRP8/14 levels correlated with both S-MRP8/14 (*r* = 0.324) and P-MRP8/14 (*r* = 0.580) levels.

**Table 1:** Demographic characteristics of the patients with juvenile idiopathic arthritis at the time of diagnosis and 1 year after the diagnosis

	Number of patients (%)			
Female sex, n (%)	62 (71)			
HLA-B27 positivity, n (%)	23 (26)			
Antinuclear antibody positivity, titer > or = 160, n (%)	41 (47)			
JIA subtype, n (%)				
Oligoarthritis, persistent	29 (33)			
Oligoarthritis, extended	4 (4.5)			
Polyarthritis, seronegative	43 (49)			
Polyarthritis, seropositive	3 (3.5)			
Enthesitis-related arthritis	8 (9)			
Psoriatic arthritis	1 (1)			
	**At diagnosis**		**At one year**	
	Median (IQR)	Mean (SD)	Median (IQR)	mean (SD)
Age at diagnosis, years	5.8 (4.7–9.6)	6.6 (4.3)		
Duration of arthritis symptoms at diagnosis, days	87 (47–185.5)	179 (276)		
Active joint count	3 (1–8)	7 (9)	0 (0–1)	1 (4)
Physician’s global assessment, VAS 0 to 100 mm	21 (11–36)	25 (18)	2 (0–8)	6 (11)
Patient’s/parent’s assessment of global well-being, VAS 0 to 100 mm	16 (5–35)	22 (20)	3 (0–13.3)	9 (13)
CHAQ^c^	0 (0–0.38)	0.2 (0.4)	0 (0–0.13)	0.1 (0.2)
Erytrocyte sedimentation rate, mm/h	13 (7–28)	20 (21)	6 (3–8)	8 (9)
C-reactive protein, mg/l	2 (2–17)	14 (28)	2 (2–2)	5 (19)
Leucocytes E9/l	7.4 (6.1–9.7)	8.1 (2.8)	5.8 (4.8–7.7)	6.3 (2.0)
Neutrophils E9/l	3.8 (2.8–5.1)	4.2 (2.0)	2.5 (2.0–3.7)	2.8 (1.2)
MRP8/14^d^ in serum, ng/ml	252 (180–317)	257 (115)	174 (113–244)	183 (103)
MRP8/14 in plasma, ng/ml	192 (100–344)	229 (168)	77 (100–136)	99 (91)
MRP8/14 in synovial fluid, ng/ml	1304 (993–1500)	1292 (409)		

Data are either median (IQR), mean (SD) or n (%) where indicated. HLA-B27 Human leucocyte antigen B27, VAS Visual analogue scale, CHAQ Childhood health assessment questionnaire, MRP8/14 Myeloid-related protein 8/14

### Disease course

**Fig. 2:**
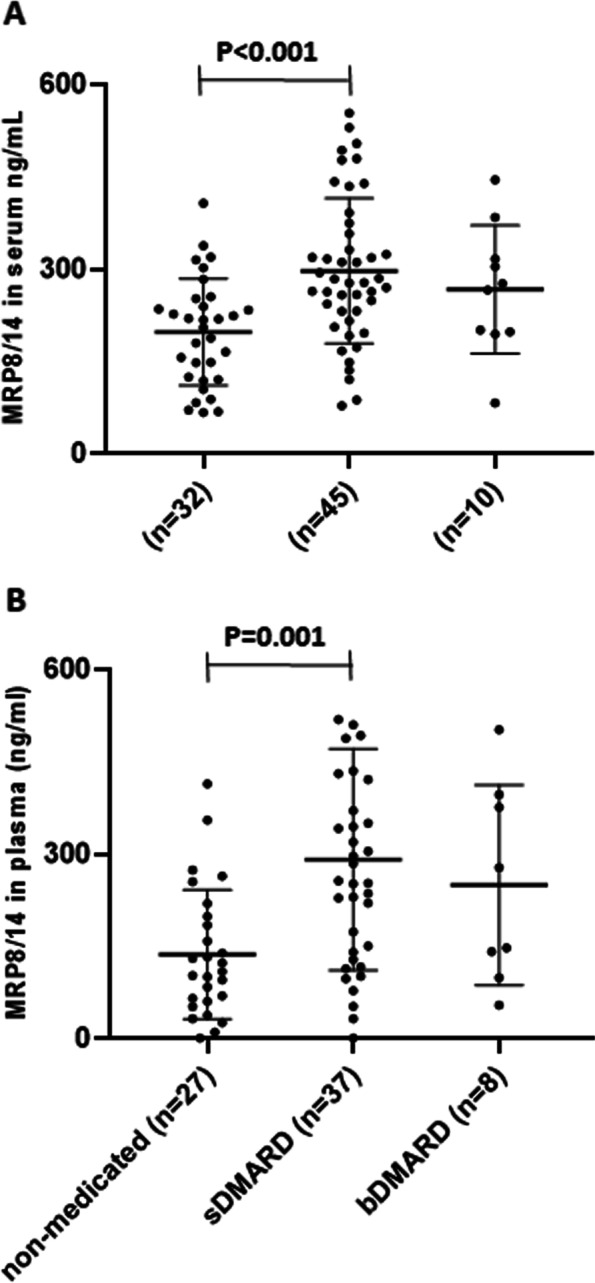
Myeloid-related protein 8/14 levels in the juvenile idiopathic arthritis patients according to need for treatment. Myeloid-related protein (MRP) 8/14 measured at JIA diagnosis in serum (S-MRP8/14) (**A**) and in plasma (P-MRP8/14) (**B**) in the treatment groups at 1 year after the diagnosis: JIA patients without DMARDs, on synthetic DMARDs (sDMARDs), and on biological DMARDs (bDMARDs). Each symbol represents the value of a given parameter for an individual patient. Results are given in terms of mean and standard deviation (SD)

Approximately 1 year (median 372 days) after diagnosis, 37% (*n* = 32) of the JIA patients did not use any DMARDs, 52% (*n* = 45) were on synthetic DMARDs (sDMARDs), and 11% (*n* = 10) were on biological DMARDs (bDMARDs). Baseline S-MRP8/14 levels were significantly higher in sDMARD users than in non-medicated patients [mean (SD) 298 ng/ml (118) vs. 198 ng/ml (87); *p* < 0.001], as were P-MRP8/14 levels [mean 291 ng/ml (180) vs. 137 ng/ml (105); *p* = 0.001] (Fig. 2.). There were no differences in S-MRP8/14 or P-MRP8/14 levels among other groups. MRP8/14 levels in blood were significantly lower after 1 year treatment, compared with baseline values [mean (SD) 184 ng/ml (115) vs. 257 ng/ml (103) in serum and 98 ng/ml (92) vs. 229 ng/ml (170) in plasma, *p* < 0.001 for both]. In 57% (*n* = 62) of the patients, the number of active joints was zero at that time.

**Fig. 3:**
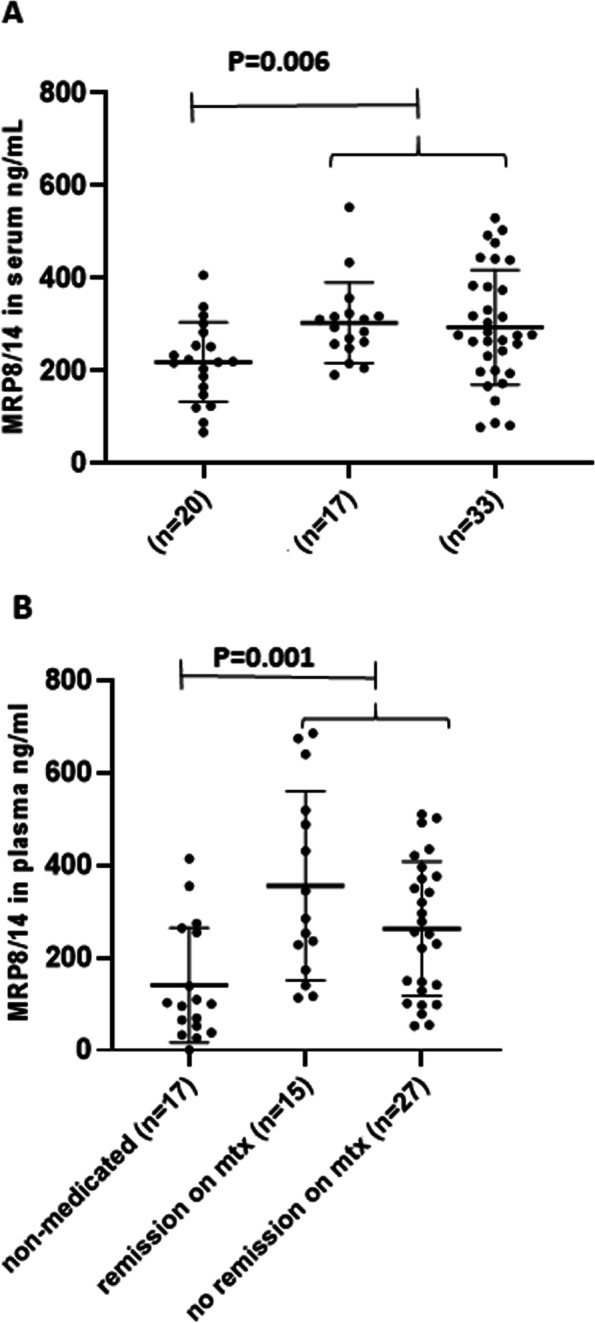
Myeloid-related protein 8/14 levels according to methotrexate treatment and outcome at 1 year. Myeloid-related protein (MRP) 8/14 measured at JIA diagnosis in serum (S-MRP8/14) (**A**) and in plasma (P-MRP8/14) (**B**) in the treatment groups according to methotrexate (MTX) treatment and remission at 1 year after the diagnosis: JIA patients without DMARDs, in remission on MTX, and no remission on MTX. Each symbol represents the value of a given parameter for an individual patient. Results are given in terms of mean and standard deviation (SD)

Fifty patients started MTX during the first 8 months after diagnosis. Of these patients, 34% (*n* = 17) achieved remission on medication within 1.5 years after diagnosis. Baseline MRP8/14 levels were significantly lower in patients who attained long lasting inactive status without medication for at least 6 months than in those who started MTX [serum: mean 219 ng/ml (86) vs 298 ng/ml (112); *P* = 0.006; plasma: 141 ng/ml (124) vs 296 ng/ml (172); *P* = 0.001] (Fig. 3.). There was no difference in MRP8/14 levels between patients who achieved remission on MTX and those who did not.

### Disease activity at disease onset

The association between the PGA and laboratory parameters was quantified using linear regression (Table 2). In univariate analysis ESR, CRP, leukocytes, S-MRP8/14, and P-MRP8/14 were related to PGA, whereas the association between neutrophils and PGA was not statistically significant. In multivariate analyses, we combined either P-MRP8/14 or S-MRP8/14 with the other laboratory parameters separately. The most effective predictive variable for PGA was P-MRP8/14 [adjusted β 0.07 (95% CI 0.03–0.10; *p* < 0.001)], whereas S-MRP8/14 was no longer significant.

**Table 2:** Linear regression analyzes for the contribution of the laboratory parameters on PGA in VAS (0-100 mm) in all the 87 juvenile idiopathic arthritis patients

Analysis	Laboratory parameters	Crude β	95% CI	*P*-value
Univariate analysis				
	C-reactive protein mg/l	0.34	0.23, 0.46	< 0.001
	ESR mm/h	0.36	0.20, 0.52	< 0.001
	Leucocytes E9/l	1.34	0.01, 2.66	0.048
	Neutrophils E9/l	1.85	−0.27, 3.98	0.09
	MRP8/14 in plasma ng/ml	0.07	0.06, 0.09	< 0.001
	MRP8/14 in serum ng/ml	0.06	0.03, 0.09	< 0.001
Multivariate analysis with MRP8/14 in plasma		**Adjusted β**		
	C-reactive protein mg/l	0.16	−0.02, 0.34	0.08
	ESR mm/h	−0.09	−0.34, 0.15	0.46
	Leucocytes E9/l	0.01	−2.12, 2.13	0.99
	Neutrophils E9/l	−0.31	−3.65, 3.03	0.85
	MRP8/14 in plasma ng/ml	0.07	0.03, 0.10	< 0.001
Multivariate analysis with MRP8/14 in serum		**Adjusted β**		
	C-reactive protein mg/L	0.29	0.11, 0.47	0.002
	ESR mm/h	0.05	−0.21, 0.31	0.69
	Leucocytes E9/L	0.59	−1.56, 2.75	0.58
	Neutrophils E9/L	−0.75	−3.9, 2.42	0.64
	MRP8/14 in serum ng/mL	0.01	−0.03, 0.05	0.56

β regression coefficient, CI confidence interval, ESR Erythrocyte sedimentation rate, MRP8/14 Myeloid-related protein 8/14, PGA Physician’s global assessment of disease activity, VAS visual analog scale

Because the number of patients with serum (*n* = 87) and plasma (*n* = 72) samples was different, we compared the MRP levels between those 15 patients who lacked plasma samples and those 72 with both serum and plasma samples, but we did not find statistically significant differences. We also performed all the same linear regression analyzes for those 72 JIA patients with both serum and plasma samples, but the result was same as in the whole 87 patient population (data not shown).

## Discussion

In this real-world cohort, we show the potential of blood MRP8/14 levels to predict the disease course of JIA for 1.5 years after diagnosis. In addition, we showed for the first time that MRP8/14 measured in plasma might be superior to that measured in serum when assessing disease activity in newly diagnosed JIA patients.

To predict the disease course, we demonstrated that blood MRP8/14 levels in newly-diagnosed DMARD-naive JIA patients were associated with the need for medication later. One third of our JIA patients were without DMARDs 1 year after the diagnosis, which is in line with the proportion of patients with the less aggressive oligoarticular JIA subtype in this study. Patients who were using sDMARDs at 1 year had higher blood MRP8/14 levels at the baseline compared to non-medicated patients, reflecting higher levels of inflammation. MRP8/14 levels in patients who used bDMARDs were higher than levels in non-medicated patients, but the difference was not statistically significant. This could be explained by the small number of the bDMARD users.

The potential role of MRP8/14 in prognostic evaluation emerged when comparing the blood MRP8/14 levels in patients who achieved clinically inactive disease status while off medication for at least 6 months during the 1.5-year follow-up period and those who started MTX treatment, regardless of treatment response. Lower levels were associated with sustainable inactive disease without systemic medication. The MRP8/14 biomarkers and their relationships to outcome within the first year after JIA diagnosis were also studied in the German Inception Cohort of Newly Diagnosed Patients with JIA (ICON-JIA) [18]. In contrast with our results, they found no association between MRP8/14 levels measured at baseline and disease activity (cJADAS ≤1 or active joint count < 1) at 12 months. However, their cohort differed from ours in that a large number of their patients (156 of 212) were treated with sDMARDs, and their cohort consisted of patients with diagnosed with JIA recently (less than 12 months before inclusion). They also divided the material more roughly than we did, as they included only two groups: active and inactive disease. In our study, disease activity was assessed more accurately, using a continuous variable, PGA. Our observation of the role of MRP8/14 as a prognostic tool is notable because of the importance of attempting to identify patients who may not need systemic medication to avoid exposing them to potential treatment side effects, as well as identifying patients who require urgent treatment interventions.

We were unable to replicate the previously observed associations in JIA patients between increased pre-treatment MRP8/14 levels and good responses to systemic drug therapy [12, 16, 17, 23]. Our results are in line with Barendregt et al.’s recent study, in which the researchers found no difference in baseline MRP8/14 levels between JIA patients who responded to treatment and those who did not [20]. In our JIA population, more than half of the patients started MTX therapy within 8 months of diagnosis. Thus, the duration of the sampling to the start of medication varied between the patients, and inflammatory activity might have changed during that period. This could be one explanation. Another might be that in our real-world cohort, we set the endpoint as remission on MTX, which is a substantial target to attain. Our study population might also reflect real-world situations more than the earlier studies did because we included all the consecutive patients with new-onset arthritis from a single hospital district area, including patients with very low disease activity. The preliminary core criteria for pediatric arthritis used in many other studies [12, 16, 17, 23] are insufficient in practice when achieving long-term remission should be the goal.

We analyzed associations between the PGA and laboratory markers in the univariate model and found relation between the PGA and P-MRP8/14, S-MRP8/14, CRP and ESR (Table 2). Recent studies have also shown an association between MRP8/14 and disease activity markers such as ESR [12, 24] and CRP [16], but only a weak correlation with a physician’s visual analog scale (VAS) [16], or a weak to no correlation with the number of active joints [12, 16]. The association with PGA and calprotectin was obtained in a cohort of rheumatoid arthritis patients in which calprotectin correlated more strongly with the PGA than with other clinical parameters (i.e., swollen or tender joints, patient’s global VAS) and was more strongly associated with calprotectin in plasma than in serum [21]. We investigated the effect of combining laboratory markers and S-MRP8/14 or P-MRP8/14 on the PGA using multivariate linear regression. We also demonstrated that a more effective set was to combine the laboratory markers with P-MRP8/14 rather than S-MRP8/14. When combining CRP and P-MRP8/14, it seemed that P-MRP8/14 might be a better marker of disease activity than CRP in newly-onset JIA (Table 2). In S-MRP8/14, that kind of behavior was not observed. This highlights the utility of P-MRP8/14 as a tool for disease activity in patients with newly-onset JIA. Moreover, in a recent study, La et al. found that S-MRP8/14 has more specificity than CRP does as a diagnostic tool and marker of disease activity for JIA [25].

Clinicians assess disease activity and evaluate patients’ condition at each visit to the rheumatology clinic. Laboratory parameters constitute one evaluation tool, but they generally do not work well for overall assessment. In our study, we assessed the patients’ overall clinical condition using the PGA as assessed on a VAS. The PGA is a general assessment of overall disease activity that can be performed easily in everyday practice. It involves subjective opinion of a clinician, and it does not require any knowledge of scoring methods. PGA estimation has been demonstrated to be a more responsive outcome measure in children with JIA than other variables used in clinical trials of JIA [26, 27]. Although there is no accurate score for this parameter, Falcone et al. [28] established a good inter-observer agreement on the PGA with a wide spectrum of disease activity and severity among JIA patients. In our cohort, we tested inter-observer agreement by defining the PGA while reading the patients’ medical records. The estimation of disease activity was quite similar between the physicians in our unit (PK and PV). A substantial limitation in our analyses was that we studied the association of MRP8/14 only with the PGA and not also with the Juvenile Arthritis Disease Activity Score (JADAS) [29]. This was the case because the patient/parent assessment of global well-being at the time of diagnosis was only available for 60% of JIA patients.

Our study has certain limitations in addition to those above. We had serum samples from all of the patients, but plasma samples from only 72 out of 87 (82%). Because of that, we also repeated our analyzes in those patients (*n* = 72) who had both serum and plasma samples and found no significant differences in results compared to the entire JIA patient data.

Most recent JIA studies focusing on MRP8/14 were assayed using serum samples [7–9, 11–14, 16, 17]. Considerably fewer studies used plasma samples [10, 23, 30, 31]. To our knowledge, this population-based study is the first to compare MRP8/14 between serum and plasma samples in JIA. Our finding regarding the superiority of assaying of P-MRP8/14 in the cohort of JIA patients is in line with Nordal et al.’s findings regarding adult rheumatoid arthritis patients [21]. As in that study, we also identified lower concentrations of P-MRP8/14 than S-MRP8/14. Nordal et al. assumed that this might be due to the increased in vitro release of MRP8/14 from activated neutrophils during the handling of blood for serum sampling. This can also lead to incorrectly high levels of S-MRP8/14 in patients with mild disease. This observation would partly explain the superiority of plasma samples compared to serum samples, as the former are more stable during handling, and the risks of artifacts are lower.

Our prospective real-world study of new-onset treatment-naïve JIA patients, demonstrates the potential of MRP8/14 in the prediction of the disease course. When we have the means to identify patients with an aggressive disease course, even at the time of diagnosis, we can quickly target them with medical interventions probably avoid subsequent consequences. In addition, patients with a mild disease course can avoid exposure to potentially harmful medication side effects.

## Conclusions

In summary, at JIA diagnosis, MRP8/14 blood levels predict the course of the disease and the need for systemic medication later. Based on our results, the measurement of MRP8/14 levels in plasma might be better than the measurement in serum when assessing disease activity in JIA. More studies are needed on the use of biomarkers as tools for predicting disease course among JIA patients in real-world treatment settings, and serum and plasma MRP8 /14 should be studied and compared in another JIA population.

## Data Availability

The data and materials used in this study can be made available on request.

## Abbreviations

ACR: American College of Rheumatology
CHAQ: Childhood Health Assessment Questionnaire
CRP: C-reactive protein
DMARD: Disease-modifying antirheumatic drugs
ELISA: Enzyme-linked immunosorbent assay
ILAR: International League of Associations for Rheumatology
JADAS: Juvenile Arthritis Disease Activity Score
JIA: Juvenile idiopathic arthritis
MTX: Methotrexate
MRP8/14: Myeloid-related protein 8/14
PGA: Physician’s global assessment of disease activity
VAS: Visual analog scale
